# Evaluating the Feasibility of Employing Dynamic Membranes for the Direct Filtration of Municipal Wastewater

**DOI:** 10.3390/membranes12101013

**Published:** 2022-10-19

**Authors:** Pau Sanchis-Perucho, Daniel Aguado, José Ferrer, Aurora Seco, Ángel Robles

**Affiliations:** 1 CALAGUA—Unidad Mixta UV-UPV, Departament d’Enginyeria Química, Universitat de València, 46100 Burjassot, Spain; 2 CALAGUA—Unidad Mixta UV-UPV, Institut Universitari d’Investigació d’Enginyeria de l’Aigua i Medi Ambient–IIAMA, Universitat Politècnica de Valencia, 46022 Valencia, Spain

**Keywords:** direct membrane filtration, dynamic membranes, resource recovery, municipal wastewater treatment

## Abstract

The aim of this study was to assess the feasibility of using dynamic membranes for direct filtration of municipal wastewater. The influence of different alternative supporting materials (one or two layers of flat open monofilament woven polyamide meshes with 1 or 5 µm of pore size) was studied. A stable short-term self-forming DM was achieved (from some hours to 3 days) regardless of the supporting material used, producing relatively similar permeate qualities (total suspended solids, chemical oxygen demand, total nitrogen, total phosphorous and turbidity of 67–88 mg L^−1^, 155–186 mg L^−1^, 48.7–50.4 mg L^−1^, 4.7–4.9 mg L^−1^, and 167–174 NTU, respectively). A DM permeability loss rate of from 5.21 to 10.03 LMH bar^−1^ day^−1^ was obtained, which depended on the supporting material used. Unfortunately, the preliminary energy, carbon footprint, and economic evaluations performed showed that although DMs obtain higher pollutant captures than conventional treatments (primary settler), the benefits are not enough to justify their use for treating average municipal wastewater. However, this alternative scheme could be suitable for treating higher-loaded MWW with a higher fraction of organic matter in the non-settleable solids.

## 1. Introduction

The current economic models based on non-renewable resources are now showing their limitations for long-term sustained development. All the estimates forecast the increasing scarcity of important essential resources, such as fresh water, energy and nutrients [1]. Finding alternatives to fossil fuels to produce energy is also essential to minimize climate change. Numerous experts have announced an urgent need to adapt our consumption to circular economy models to achieve sustainable human development [2]. Following this approach, municipal wastewater (MWW) treatment is now experiencing an important paradigm shift. In fact, MWW could not only be a source of recycled water, but also a source of energy and basic nutrients (nitrogen and phosphorus) [3]. Unfortunately, the current municipal wastewater treatment plants (MWWTP) are unable to completely recover all the potential resources contained in MWW. Conventional activated sludge (CAS) systems, which are the core of MWW treatment, fail to recover the potential energy in influent organic matter by removing it via biological oxidation only. The nutrients present are also usually wasted, removing the ammonium concentration by nitrification/denitrification and precipitating the phosphate by chemicals that disable them for reuse. CAS treatments also demand a large amount of energy for removing organics from sewage, representing around 30–60% of the total MWWTP energy requirements [4]. Alternative treatment schemes thus need to be developed to take advantage of all the potential resources from MWW and change the former conception of MWWTPs to new resource recovery facilities.

Several alternatives have been developed to achieve sustainable MWW treatments, anaerobic membrane bioreactor technology being one of most attractive due to the possibility of converting influent organic matter into methane [5]. However, the considerable financial investment required to adapt current installations to alternative systems hinders their introduction. In this context, direct sewage filtration, defined in the literature as direct membrane filtration (DMF), has recently emerged as an interesting alternative for upgrading the energy efficiency and resource recovery of current MWWTPs [6]. This strategy consists of implementing a membrane system before the CAS process to capture the material suspended in the influent. Thanks to this previous filtration, a large amount of influent organics (the complete particulate fraction) would be recovered before CAS treatment, dramatically reducing the aeration demands of the process and MWW energy requirements. The organic matter captured in the membrane tank could be used to produce methane via anaerobic digestion (AD), enhancing the overall process energy balance even further. A significant portion of influent nutrients (suspended fraction) could also be recovered in the membrane tank’s concentrated sludge, allowing the DMF to enhance the overall MWWTP resource recovery potential without seriously modifying the installations. MF and UF membranes have been extensively tested for this and have obtained promising results [7]. Unfortunately, numerous studies have reported severe membrane fouling when operating these membranes with untreated MWW [8,9], which compromises their feasibility by requiring energy-consuming membrane fouling control strategies during filtration. Additionally, low/moderate permeate fluxes have been recommended when operating these systems [10], sharply increasing the initial investment in membranes for full-scale implementations. This suggests that membrane systems with lower fouling propensities, such as dynamic membranes (DM), could be an interesting alternative to conventional membrane systems to carry out DMF treatment schemes.

DMs consist of the formation of a stable cake layer on a low filtration-resistance supporting material, which is the main filtration element [11]. Thanks to removing the intrinsic filtration resistance of conventional membranes, higher permeabilities can be achieved during filtration, which can be controlled by acting on the thickness and density of the cake layer [12]. Membrane fouling thus changes its paradigm, in this case by playing a partially beneficial role that can be easily controlled by physical low energy cleaning [12]. In addition, the supporting structures are generally made of low-cost materials, such as woven meshes or filter-cloths, which have a significantly lower acquisition and/or replacement cost than conventional membrane modules [13]. However, despite their potential benefits, DMs involve different issues that need to be addressed. Significantly worse permeate qualities can be expected when using DMs instead of MF or UF, since the cake layer formed has a less homogeneous structure with higher porosity than commercial membranes. As the cake layer mainly controls filtration performance, the permeate generated by DMs may be unstable to some degree, changing according to the characteristics of the DM formed during filtration. The formation of a stable DM can also represent difficulty in some cases, since its formation strongly depends on the characteristics and concentration of the material suspended in the influent and its interaction with the supporting structure. In this respect, two different DMs can be distinguished, depending on whether the filtering cake layer developed on the supporting structure is self-forming or pre-coated [11,14]. Self-forming DMs are created when the filtering cake layer consists of direct deposits of particulate material on the supporting structure during filtration, while pre-coated DMs consist of a previously stable structure on which the influent particulate material can be deposited to form the filtering cake layer. Pre-coated DMs are in theory less advantageous, since they require auxiliary chemical dosing, which increases their operating costs [14]. Selecting a proper supporting material to enable the short-term self-formation of the DM when possible can thus be a key issue for boosting the feasibility of this technology.

Few studies to date have assessed the feasibility of DMs for the DMF strategy and further studies are required to prove their potential. Real MWW instead of synthetic solutions need to be studied to consider more realistic interactions between the influent particulate material and the supporting structure. Larger membrane areas than those used in laboratory-scale studies also need to be tested to consider possible hydrodynamic issues that could hinder DM formation during filtration of difficult sludge recovery in full-scale applications. The aim of this work was thus to evaluate the feasibility of a DM pilot plant (2 m^2^ filtration membrane area) for directly filtering the real influent of a full-scale MWWTP. The influence of different alternative supporting materials was studied (one or two layers of 1 or 5 µm pore size flat woven polyamide open monofilament meshes) to assess the effect on the self-forming DM capacity, filtration performance, and permeate quality. The proposed alternative potential was evaluated by performing a preliminary energy, economic, and carbon footprint balance.

## 2. Materials and Methods

### 2.1. Pilot Plant

**Figure 1:**
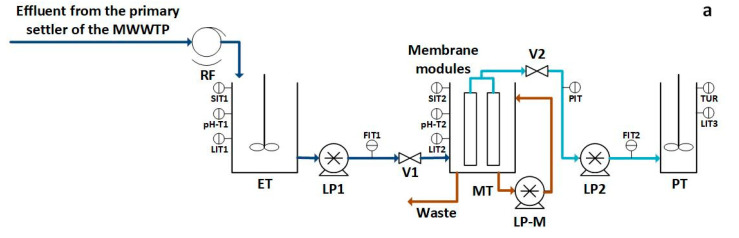

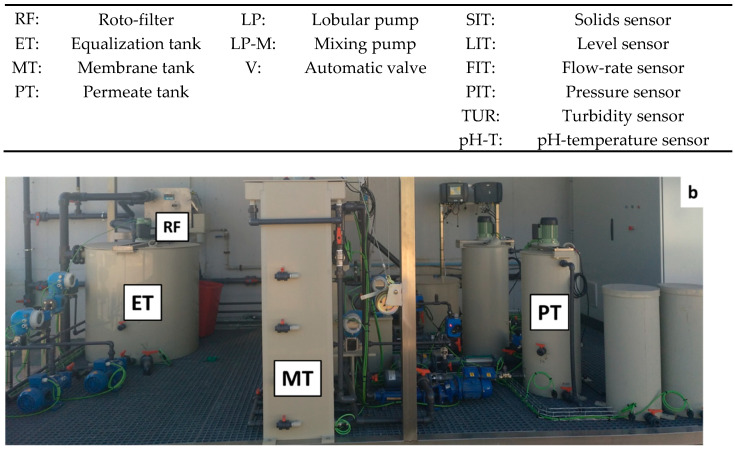
DM pilot plant: (**a**) schematic diagram and (**b**) picture.

The DM pilot plant used in this study consisted of a 190 L working volume membrane tank equipped with two flat submerged membrane modules. Each membrane module consisted of a 1-m high × 0.5-m wide membrane frame that supported the DM supporting materials for filtration (average pore size and number of layers depending on the experimental period). The membrane frames allowed the attachment of two supporting materials (one on each module face), recovering the generated permeate in the interstitial space. The total filtration membrane area of the modules was 2 m^2^. A large-pore woven steel mesh was added under each textile layer to stiffen the supporting material during filtration. The pilot plant was operated continuously at a permeate flux of 15 LMH, performing infinite filtration–relaxation cycles. Filtration lasted for 180 s, while 60 s were set for the relaxation stages, achieving a filtration-to-relaxation ratio of 3:1. Filtration was performed by vacuum using a lobular pump (PCM, M Series, EcoMoineau™, Milano, Italy). The membrane module content was continuously mixed by a similar lobular pump to ensure homogeneity. DM filtration was at a constant total suspended solids (TSS) concentration of about 2.1 g L^−1^ during all experimental periods. Membrane waste was evacuated continuously at a flow rate of 3.3 L h^−1^ to maintain TSS concentration, giving a membrane tank solids retention time (SRT) of 2.4 days. The raw influent MWW was pre-treated with a 0.5 mm screen size roto filter (PAM 270/500, Procesos Auto-Mecanizados, Alicante, Spain) and stored in 745 L working volume equalization tank (1.4 h hydraulic retention time (HRT)) for continuous feeding of the membrane module. A lobular pump (PCM, M series, EcoMoineau™, Milano, Italy) was used to feed the DM membrane module according to filtration requirements. Figure 1 shows a schematic diagram and picture of the pilot plant. Further information on this system can be found in Sanchis-Perucho et al. [15].

### 2.2. Influent and Experimental Plan

Raw MWW (after classic pre-treatment by screening and sieving followed by desanding and degreasing) from the full-scale *Conca del Carraixet* WWTP (Alboraya, Spain) was used as pilot plant influent. The main characteristics of this MWW can be found in Table 1. Four textile-mesh-based alternatives (combinations of two pore sizes in simple or double layers) were evaluated as possible supporting materials for self-forming the DM (see Table 2). Flat open monofilament woven polyamide meshes (NITEX^®^, SEFAR) were used in all cases. Physical cleaning was performed by brushing the membrane surface with tap water as required.

### 2.3. Analytical Methods and Calculations

The pilot plant influent-generated permeate and waste were sampled twice a week. TSS, chemical oxygen demand (COD), total nitrogen (TN), and total phosphorous (TP) were determined according to standard methods [16]. A laser granularity distribution analyzer with a detector ranging from 0.01 to 1000 µm (Mastersizer 2000, Malvern, UK) was used to evaluate the particle size distribution of the fresh influent fed to the membrane tank. DM performance was evaluated based on its permeability evolution. 20 °C-standardized permeability (*K*_20_) was calculated according to the following expression, which can be deduced from [15]:

(1) $$K_{20}=\frac{J_{T}\cdot e^{-0.0239\;\left(T-20\right)}}{TMP_{ave}}$$

where *J_T_* represents the recorded permeate flux, *T* is the temperature and *TMP_ave_* is the average transmembrane pressure recorded during each filtration cycle.

A preliminary evaluation was made of process energy, carbon footprint, and economic costs. Since this alternative scheme focused on upgrading current MWWTPs, general water and sludge treatment equipment was not contemplated. For the energy balance, the energy demands of the permeate pumping and mixing and the potential energy recovery achieved from the organic matter captured by the DM were considered. Accordingly, the carbon footprint analysis only considered the energy required by the process. The economic balance considered both capital expenses (CAPEX) and operating expenses (OPEX). For the CAPEX, only the cost of the supporting material acquisition was considered, while the OPEX included the energy demands of the process and supporting material replacements. An average treatment volumetric flow rate of 36,625 m^3^ d^−1^ was considered for all the calculations. This flow rate coincides with that of the MWWTP where the pilot plant operated.

Equipment energy demands were calculated according to their appropriate theoretical equations (Equations (2) and (3)) [17]:

(2) $$P_{P}=\frac{Q_{P}\cdot TMP_{ave}}{\eta_{P}}$$

where *P_P_* is the filtration permeate pump power requirements (W), *Q_P_* is the pump volumetric flow rate (m^3^ s^−1^), *TMP_ave_* is the average transmembrane pressure during filtration (Pa), and *η_P_* is the pump efficiency, the value of which was set in 0.65 in this study.

(3) $$P_{M}=Q_{M}\cdot \rho \cdot g\cdot \frac{\left\{\left[{\left(\frac{\left(L+L_{eq}\right)\cdot f\cdot v^{2}}{D\cdot 2\cdot g}\right)}_{A}+{\left(\frac{\left(L+L_{eq}\right)\cdot f\cdot v^{2}}{D\cdot 2\cdot g}\right)}_{I}\right]+\left[z_{1}-z_{2}\right]\right\}}{\eta_{P}}$$

where *P_M_* is the mixing pump energy requirements (W), *Q_M_* is the mixing volumetric flow rate (m^3^ s^−1^), *ρ* is the mixed liquor density (Kg m^−3^), *g* is the acceleration of gravity (m s^−2^), *L* and *L_eq_* are the pipe length and equivalent pipe length (m), respectively, *v* is the liquor velocity (m s^−1^), *f* is the friction factor, *D* is the pipe diameter, and (*z*_1_ − *z*_2_) is the height difference (m). On the other hand, the following expression was used to estimate the energy production when transforming the recovered organic matter into biogas [15]:

(4) $$E_{R}=COD_{R}\cdot Y^{CH4}\cdot CV_{CH4}\cdot \eta_{CHP}$$

where *E_R_* is the energy recovery (kWh m^−3^), *COD_R_* is the recovered COD concentration in the membrane module (kg m^−3^), *Y*^*CH*4^ is the theoretical anaerobic methane yield of MWW sludge (3.5·10^−4^ m^3^ of methane per kg of COD), *CV*_*CH*4_ is the methane calorific power (9.13 kWh per m^3^ of methane), and *η_CHP_* is the CHP system methane electricity generation efficiency. A *η_CHP_* of 35% was used considering the different CHP technologies currently available [18].

For the carbon footprint calculations, a global warming potential (GWP) of 0.36 kg CO_2_-eq per kWh of consumed energy was considered, in accordance with the energy mix GHG emissions ratio expressed in EcoInvent database [19]. Concerning energy and process costs, €0.20 per kWh was estimated for the electricity cost according to current Spanish high-voltage electricity rates [20], while the supporting material acquisition cost was estimated at €0.7 per m^2^ of membrane area, according to Millanar-Marfa et al. [21]. Although some studies assume that no supporting material replacements will be required [21], we estimated a supporting material lifespan of 10 years.

## 3. Results and Discussions

### 3.1. DM Self-Forming Capacity and Filtration Performance

**Figure 2:**
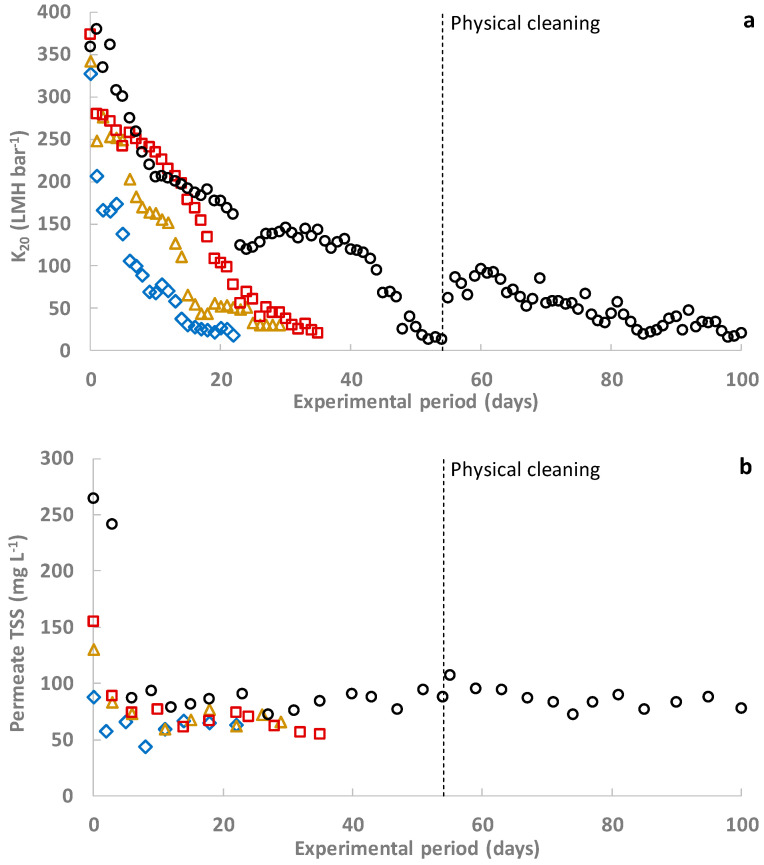
DM performance regarding the employed supporting material: (**a**) permeability evolution and (**b**) TSS concentration in the permeate. 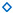 Two layers with 1 µm of pore size, 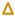 one layer with 1 µm of pore size, 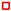 two layers with 5 µm of pore size, 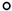 one layer with 5 µm of pore size.

Figure 2a shows the permeability evolution of the self-formed DM during each experimental period. A slightly shorter self-forming time was achieved as the supporting material pore size was reduced. Similarly, the use of additional supporting material layers also entailed shorter DM self-forming periods. In both cases, the reduced DM self-forming time was due to the higher solids retention capacity achieved in the first days of filtration when reducing the supporting material pore size or adding additional layers (see Figure 2b). Slightly lower permeability of the virgin supporting textile woven mesh was found as pore size was reduced and the layers were raised, indicating that the supporting material presented higher filtration resistance, which helped to retain the influent particulate pollutants (see Figure 2a). Since more particles were retained in the supporting mesh, more material was used to create a preliminary cake layer on the woven textile mesh and promote the formation of a stable DM. Unfortunately, although lower self-forming times were obtained when increasing the supporting material filtration resistance, the enhanced solids retention capacity also entailed a sharp reduction of DM permeability as filtration advanced (see Figure 2a). Permeability was reduced by 90% after 14, 24, 32, and 50 days in Experiments 1 to 4. However, the TSS concentration captured during filtration reached a pseudo-steady state after a stable DM was formed (ranging from several hours to 3 days, depending on the experimental period; see Figure 2b), and did not increase despite the lower DM permeability. According to these results, the drop in DM permeability was related to the quicker increase of DM thickness when using supporting materials with a higher filtration resistance, and relevant short-term alterations of the DM structure are not expected. Since no relevant reduction of the self-forming time was achieved as the supporting material filtration resistance was increased, the use of a 5 µm pore size single layer was considered the most suitable material to extend the filtration lifespan.

The DM formed in Exp. 4 was cleaned by brushing the surface with tap water when the permeability dropped under 50 LMH bar^−1^. This reduced the DM thickness, recovering a great part of the original supporting material permeability without compromising the DM solids capture capacity (see Figure 2). The filtration lifespan was then extended for about a further 50 days, although at significantly lower permeability (average permeability of about 55 LMH bar^−1^ was obtained after the physical cleaning, in contrast with the 141 LMH bar^−1^ achieved in the first half of Exp. 4). The lower permeability achieved after the physical cleaning may have been due to deficient cleaning not having removed enough DM thickness or, as other studies have found [22], it could have been produced by internal blockage of some of the supporting material pores, which cannot be efficiently removed by physical cleaning. In any case, since low-energy physical cleaning methods were relatively effective in controlling DM thickness, applying a proper cleaning schedule would be interesting to enhance process feasibility without compromising energy demands.

**Figure 3:**
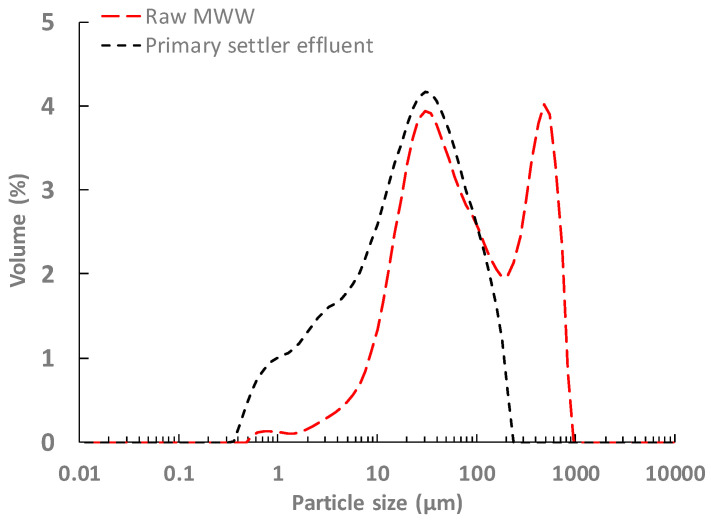
Particle size distribution of the raw MWW used in this study and the primary settler effluent filtered in Sanchis-Perucho et al. [17].

Important differences in performance were achieved between the results obtained in this study using raw MWW as influent and previous studies that used primary settler effluent [15]. Much shorter DM self-forming periods were obtained with raw MWW, allowing the larger supporting material pore size. Only a few hours were required to self-form a stable DM with raw MWW in contrast to the 17 days required with primary settler effluent as membrane tank influent (two layers of a woven polyamide mesh with 1 µm pore size in both studies). This significant difference was due to both the higher content of particulate material in the treated influent when using raw MWW and the larger average size of the influent particles. Indeed, the particles between 100 and 1000 µm increased significantly when raw MWW was used (see Figure 3), which favoured the development of a DM by promoting the formation of a preliminary cake layer on the supporting material. However, as the self-forming time was reduced, the filtration fouling growth rate increased, achieving permeability losses in the DM of between 10.03 and 5.21 LMH bar^−1^ day^−1^ (see Table 2), depending on the supporting material used in this study with the 2.27 LMH bar^−1^ day^−1^ reached when filtering primary settler effluent [15]. Then, although using raw MWW could be considered as a more suitable influent for applying DMs when filtering MWW, more energy may be required to control the DM thickness. Consequently, further studies focused on the overall process energy requirements and resource savings are required to properly determine the best influent to use (filtration energy demands according to DM permeability, applied fouling control strategies energy requirements, percentage of organic matter and nutrients captured from different influents, etc.).

### 3.2. Permeate Quality

Table 3 shows the average DM permeate quality and resource recovery during each experimental period after the DM was formed. Higher pollutant retention was obtained as the supporting material pore size was reduced and more layers were added, thus increasing the quality of the permeate. However, the benefits in permeate quality after achieving a stable DM were negligible, with only a 6% difference between the most efficient solids-capturing supporting material (two layers with 1 µm pore size) and the less efficient one (one layer with 5 µm pore size). Since there was no great difference in permeate quality, it can be concluded that no significant changes in the surface or internal DM structure can be expected in the supporting textile materials used in this study, the slightly higher retentions being due to the thicker DM formed when increasing the supporting material solids retention capacity. Similar permeate qualities have also been reported by other authors when filtering raw MWW with DMs, despite employing larger supporting material pore sizes (between 1 and 100 µm) or adding an extra suspended material component (diatomite) to boost DM formation [12,23]. In fact, the permeate produced after obtaining a stable DM seems to be relatively consistent when filtering untreated MWW, achieving similar qualities when filtering the primary settler effluent of an MWWTP [15]. Since the supporting material or influent used does not seem to significantly influence the permeate quality, the selection of the most suitable configuration should focus on reducing the filtration energy demands.

Comparing the DM permeate quality to the primary settling effluent from the *Cuenca del Carraixet* MWWTP, a significantly higher particulate material fraction was captured in the former than in the latter (73% in the DM compared to the 59% captured by primary settling; see Table 3), showing its potential as primary treatment. A higher COD content was therefore also recovered in the DM unit (64%) than in the primary settling step (57%). The lower differences achieved for COD were due to the significant concentration of COD in the influent soluble fraction, which none of the compared technologies could capture. If the soluble COD in the influent wastewater were to be negligible, the difference in COD capture between the DM and the primary settler would be higher (and similar to the existing difference in TSS). On the other hand, poor permeate qualities were obtained when comparing the results of this work with other membrane technologies used for DMF (i.e., MF and UF membranes). This could be expected, since the lower pore size of MF and UF membranes (from about 1 to 0.01 µm) can capture almost all influent particulate (and colloidal) material, achieving permeates without solids and with COD, TN, and TP concentrations of about 44–88, 46.1–48.2, and 6.44–6.45 mg L^−1^, respectively [24]. In this context, DMs cannot compete in COD recovery, although relatively similar TN and TP captures can be achieved, since the main input of these pollutants comes in the form of soluble compounds (see Table 3). In any case, since MF and UF membranes involve significantly higher costs than DMs (about €35 per m^2^ of MF and UF membranes compared to the €0.7 per m^2^ of DMs) [21,25], besides higher operating costs and fouling propensities [12], they require substantially, and possibly prohibitive, investment costs when just aiming to upgrade an existing facility. DMs can thus be an interesting alternative when targeting improving the amount of resources recovered during classic MWW treatment at low costs.

According to the results obtained, the permeate quality generated by the DM is far from meeting the European standards regarding direct reuse or discharge into water bodies, especially due to its significant nutrient content. Since a considerable fraction of the influent COD was recovered thanks to the DM treatment, the effluent produced could be treated by a CAS process, which would require less energy thanks to its reduced aeration demands. This CAS process could focus on influent nitrification/denitrification treatment while using the remnant COD, removing the phosphorous concentration by biological capture when possible, or using conventional chemicals for its precipitation. An aerobic bacterial and microalgae consortium to remove the remnant COD while capturing nutrients could be another possible alternative [26]. Since the bacterial oxygen demands would be covered by the microalgae activity, this alternative could be proposed as a low-energy treatment. Thus, although significantly worse permeate qualities can be expected when replacing MF or UF membranes by DM, a sizeable fraction of the influent COD would still be recovered via AD, alongside the ability to capture the influent nutrients by secondary alternatives.

### 3.3. Process Feasibility

**Figure 4:**
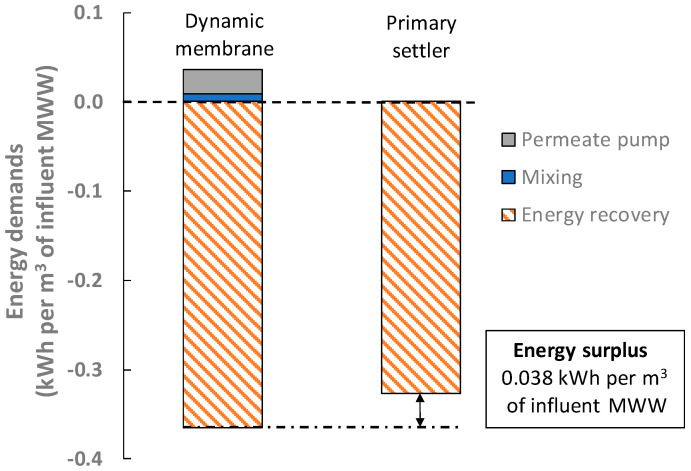
Energy recovery potential of the direct raw MWW filtration by DMs.

**Figure 5:**
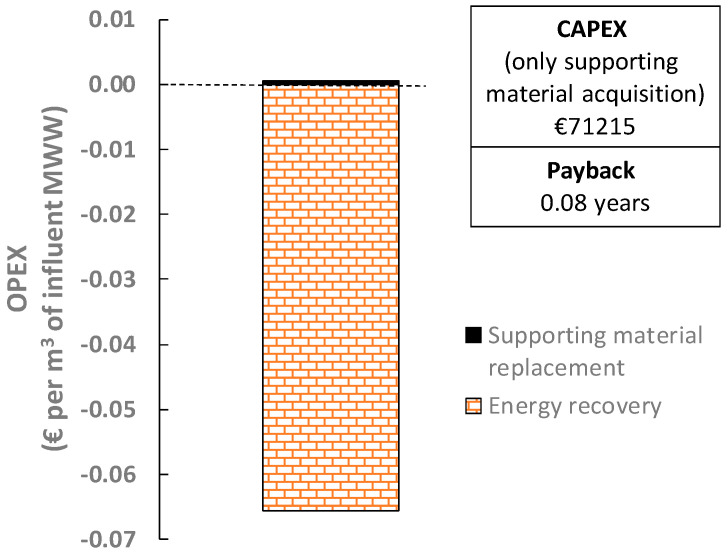
Preliminary economic potential of the direct filtration of raw MWW by DMs. CAPEX: capital expenses. OPEX: operating expenses.

To assess the feasibility of the proposed alternative, three points of view were considered: energy, economy, and the carbon footprint. Figure 4 shows the filtration energy demands together with the energy that could be potentially recovered after transforming the captured organic matter into methane via AD. Since only permeate pumping and mixing would be required when using DMs, significant energy recoveries are achieved by this process, which rise to about 0.33 kWh per m^3^ of influent MWW. Indeed, the fouling control strategies required in other membrane systems (membrane bioreactors) are the largest energy consumers [27]. Unfortunately, an insignificant enhancement on the energy recovery was achieved compared with a classic MWWTP primary settler (see Figure 4), which does not justify the use of DMs. This alternative also showed relevant carbon footprint reductions (about 0.13 kgCO_2_-eq per m^3^ of influent MWW), thanks to the organic matter captured by the DM, although again with a negligible difference over a conventional primary settler. Positive financial results were obtained for the proposed alternative, thanks to the significant amount of energy recovered, which far exceeded the replacement costs of the supporting material (see Figure 5), while the initial investment was significantly reduced due to the relatively lower cost of supporting materials than those required by other membrane systems. In fact, short payback periods can be expected; we achieved 0.08 years in the present study, with a profit of about €0.065 per m^3^ of influent MWW. However, as mentioned above, this profit is not meaningful enough when considering the amount of organic matter captured by a MWWTP primary settler, which obtains similar outcomes. Additionally, physical low-energy fouling control strategies need to be studied to improve DM permeability during continuous filtration. The environmental and economic impact of the materials and other auxiliary resources required by this alternative (e.g., membrane tanks, maintenance demands, equipment replacements, etc.) should also be considered to properly study its feasibility. The small profit made by the organic matter capture of DMs is therefore not large enough to justify its implementation when treating average MWWs, as this alternative scheme requires a higher resource recovery potential to be competitive. The alternative was found to be unsuitable for treating low/middle-pollutant-load MWWs and further studies considering high-load MWWs or similar influents are required.

**Figure 6:**
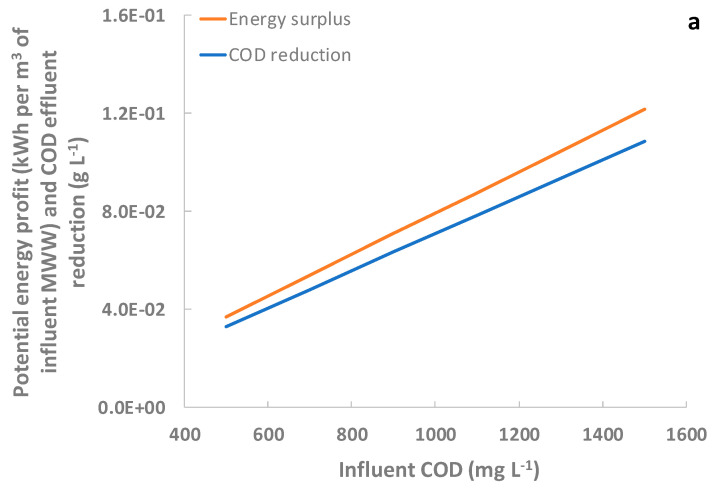

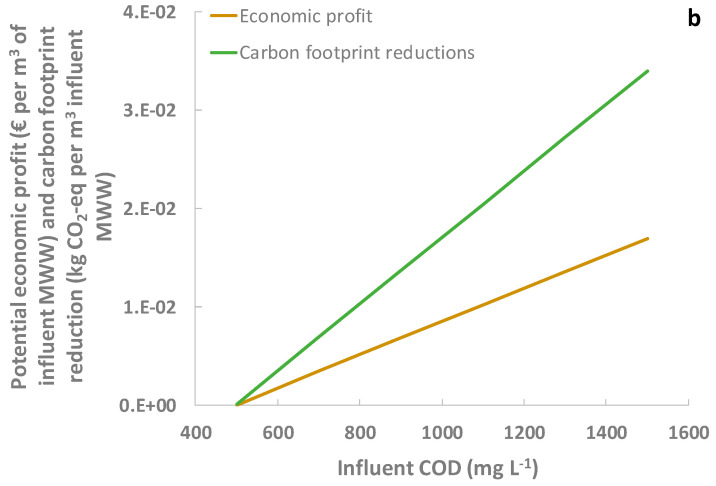
DM potential benefits compared to primary settling as influent COD increases: (**a**) energy surplus and effluent COD reductions, and (**b**) carbon footprint reductions and economic profit.

To study the potential benefits of DMs over conventional treatment schemes with heavily loaded wastewaters, the energy, carbon footprint, and financial profit of both DM filtration and primary settling were calculated for different increased COD concentrations (from 500 to 1500 mg L^−1^). The same particulate fraction captures as those obtained in this study were considered for both systems in these simulations. The surplus obtained by DM is shown in Figure 6. Larger profits can be achieved than those obtained in this study when considering a heavily-loaded MWW (COD about 1000 mg L^−1^ [28]), with an energy surplus of 0.08 kWh per m^3^ of influent MWW with DMs instead of primary settling (see Figure 6). Better carbon footprint reductions can also be obtained in this scenario, achieving a surplus reduction of 0.017 kgCO_2_-eq per m^3^ of influent MWW. It is important to highlight that these potential advantages only consider the direct benefits of higher DM COD capture. Thanks to the greater particulate COD recovered by DMs, low-loaded effluents should be treated by CAS or other secondary treatments that require a lower energy input, thanks to the reduced aeration necessities. Considering 1000 mg L^−1^ of COD in the influent, a direct financial surplus of €0.009 per m^3^ of influent MWW would be obtained by substituting the primary settler with a DM system, also reducing the amount of COD to treat in the secondary treatment by 71 mg L^−1^. This should be considered in the energy, carbon footprint, and economic balance as indirect profits. This treatment scheme could therefore be attractive for heavily loaded MWWs or industrial wastewaters with a high percentage of non-settable organic particulate material. Finally, as other authors have proposed [12,15], using coagulants to increase the average influent particulate fraction size would be an interesting strategy to increase the potential DM resource recovery even further. In a previous study using the primary settler effluent as the base [15], it was determined that relatively low coagulant dosing (10 mg L^−1^) significantly increased the COD captured by the DM (effluent COD reduction from 141 to 58 mg L^−1^) and recovered a relevant fraction of the soluble COD fraction along with all the influent phosphate. Further studies are therefore required to evaluate the advantages of dosing coagulants during raw MWW filtration when using DMs.

## 4. Conclusions

This study assessed the feasibility of treating MWW with DMs. A stable short-term self-forming DM was achieved, regardless of the supporting material used (from several hours to 3 days) thanks to the significant concentration of particulate material and the large particle size present in the raw MWW. Relatively similar permeate qualities were obtained for all the supporting materials tested, although higher permeability losses (from 5.21 to 10.03 LMH bar^−1^ day^−1^) were found as the supporting material filtration resistance increased due to the increasing DM thickness. A single-layer supporting material of 5 µm or larger pore size can be recommended to minimize DM thickness growth rate. Unfortunately, our preliminary energy, carbon footprint, and economic evaluations showed that, although DMs capture more pollutants than conventional treatments (primary settler), the benefits are not enough to justify their use with average municipal wastewater. However, this alternative scheme could be suitable for treating higher-loaded MWW with a higher fraction of organic matter in the non-settable solids.

## Figures and Tables

**Table 1:** Influent characteristics.

Parameter	Units	Mean ± SD
TSS	mg TSS L^−1^	321 ± 98
COD	mg COD L^−1^	512 ± 118
SCOD	mg COD L^−1^	63 ± 28
TN	mg N L^−1^	56.7 ± 10.8
TP	mg P L^−1^	6.4 ± 1.6
Alk	mg CaCO_3_ L^−1^	342 ± 73
pH	-	7.4 ± 0.7
Turbidity	NTU	399 ± 124

**Table 2:** Supporting material characteristics and average DM permeability losses obtained during each experimental period.

Exp.	Supporting Material Employed		Fouling Growth Rate	
	Layers	Average Pore Size (µm)	Slope (LMH bar^−1^ d^−1^)	R^2^
1	2	1	10.03	0.789
2	1	1	9.85	0.888
3	2	5	9.24	0.955
4	1	5	5.21	0.877

**Table 3:** DM permeate quality and resources captured.

Exp.	TSS		Turbidity		COD		TN		TP	
	(mg L^−1^)	(%) *	(NTU)	(%) *	(mg L^−1^)	(%) *	(mg L^−1^)	(%) *	(mg L^−1^)	(%) *
1	67	21	167	55	155	30	48.7	86	4.7	73
2	73	23	157	53	159	31	50.1	88	5.0	78
3	70	22	161	55	167	33	49.4	87	4.8	75
4	88	27	174	59	186	36	50.4	89	4.9	77
PS	132	41	-	-	218	43	-	-	-	-

* Percentage of the influent pollutant remaining in the permeate. PS: solids and organic matter captured by the primary settler of the MWWTP.

## Data Availability

Not applicable.
